# Supplementary material for: Improving population scale statistical phasing with whole-genome sequencing data
Source: PLoS Genet. 2024 Jul 3;20(7):e1011092. doi: 10.1371/journal.pgen.1011092 (PMC11251608; doi:10.1371/journal.pgen.1011092)
Supplement: S1 Algorithm — (PDF) [file pgen.1011092.s002.pdf]

# S1 Algorithm.
# Heterozygous variant extraction from VCF/BCF file.

The first step in the SAPPHIRE pipeline (step A. in Fig 9) is to extract heterozygous variants for processing as homozygous variants provide no phase information. The goal is to extract heterozygous variants with a low phasing confidence (SHAPEIT5 PP field, or MAF as a proxy) and their closest neighboring heterozygous variants. Because the closest heterozygous variants loci differ for each sample and loading the matrix composed of all genotypes at all variant loci for all samples in memory is intractable, the extraction is done by filling a bound size FIFO (first-in first-out) container for each sample while traversing the input VCF/BCF file. The extraction algorithm is given in Algorithm S1-1. The input VCF/BCF file can be seen as a matrix of genotypes, so in order to limit memory usage, the file is processed line by line (variant locus by locus). For each line, heterozygous genotypes are saved in a record along with their PP score, and a reference to the VCF line. The PP score is either provided by SHAPEIT5 or inferred from MAF. Common variants (without a PP field) are considered as if they had a score of 1.0 (100% confidence). Each of these records are put in the FIFO of the corresponding sample. Each FIFO will collect heterozygous genotypes for the sample by adding the new record at the end and discarding the oldest record. If a low-PP genotype passes the middle of the FIFO, the records are collected in an internal list. The FIFO size is a parameter of the extraction software. For example, a size of five, the default value, will collect low-PP genotypes with four neighboring heterozygous genotypes, two located before and two located after. To avoid edge cases and simplify the logic, the FIFO is pre-filled with *dummy records* that will simply not be copied to the internal list. At the end of the VCF/BCF input file the FIFOs are flushed to get possible low PP genotypes that would not have neighbors after them. Finally the internal lists of genotype records for all FIFOs are written to a SAPPHIRE binary file[1].

**Algorithm S1-1:** Extraction Algorithm

```
Input  : Single chromosome VCF/BCF file with M variant loci and N samples
         Abstracted as a genotypes[M][N] matrix, accessed line by line
Output: Binary SAPPHIRE file with extracted heterozygous variants
for i ← 0; i < M; i++ do
    for s ← 0; s < N; s++ do
        genotype ← genotypes[i][s];
        if genotype is heterozygous then
            get PP of genotype or compute it from MAF;
            genotype_record := {genotype, PP, VCFline};
            FIFO[s].put(genotype_record);
        end
    end
end
for s ← 0; s < N; s++ do
    FIFO[s].flush();
    FIFO[s].write_to_file();
end
```

The algorithmic complexity of Algorithm. S1-1 is $\mathcal{O}(MN)$ where M is the number of variant loci and N is the number of samples. Computing PP from MAF is experimental and for the moment is implemented as follows: PP is set to 1.0 when MAF is above a threshold (common variants, default value 0.1%) and arbitrarily as $0.5 + \text{MAF}/2$ for rare variants. Future works include creating a better model for PP relative to MAF, if necessary, as SAPPHIRE was designed to run on SHAPEIT5 phased data with PP field. As SAPPHIRE is open source, researchers can change the PP from MAF computation to a more suitable model for their data if needed. A MAF threshold of 0.001 with the above formula allows for very accurate phase polishing without the PP field, as shown in Fig 10.

**Algorithm S1-2:** FIFO put

```
Input  : Genotype record to add to FIFO
append genotype_record to the back of FIFO;
if middle element of FIFO is low-PP (< 0.99) then
    copy all variants in FIFO to internal list without duplicating variants already in list;
end
pop front element of FIFO;
```

**Algorithm S1-3:** FIFO flush

```
if FIFO contains low-PP not already copied to internal list then
    copy all variants in FIFO to internal list without duplicating variants already in list;
end
```

The FIFO `write_to_file()` method writes the genotype information in SAPPHIRE binary format.The binary file format allows to query the genotypes extremely quickly and makes transformations (split and merge) possible to allow distributed processing of the next stage. For example a binary file of 200k samples can be split into smaller files of 1,000 samples quasi instantly, allowing for batch processing of 1,000 samples on distributed compute nodes. The processed binary files can then be merged again after processing.

[1]The file format is described at `https://github.com/rwk-unil/sapphire/blob/main/pp_extractor/doc/Binary_Format.md`
