# Supplementary material for: Improving population scale statistical phasing with whole-genome sequencing data
Source: PLoS Genet. 2024 Jul 3;20(7):e1011092. doi: 10.1371/journal.pgen.1011092 (PMC11251608; doi:10.1371/journal.pgen.1011092)
Supplement: S2 Algorithm — (PDF) [file pgen.1011092.s003.pdf]

S2 Algorithm. Phase calling (polishing).

This is the main step of the SAPPHIRE pipeline (step B. in Fig. 9). The phase calling algorithm is given in Algorithm S2-1. As this algorithm is independent for each sample, it can be both multi-threaded on a compute node and distributed on multiple compute nodes.

Algorithm S2-1: Phase Calling (Polishing) Algorithm

```
Input  : VCF/BCF file with variant loci only, SAPPHIRE binary file with extracted heterozygous
         genotype records, a CRAM file with sequencing data for each sample (N samples).
Output: Updated SAPPHIRE binary file
Load VCF/BCF file with variant loci information (the genotype records reference these through
  their VCF line attribute);
for s ← 0; s < N; s++ do
    Load extracted genotype records for sample s from file, ignoring non SNVs;
    Create a doubly linked list list with all records;
    for all genotype records gr in list do
        Pile-up reads from CRAM whole-genome sequencing file on the variant position applying
          read_filter() // Done through the HTSLIB library;
        Collect references to reads that carry the first allele in a set in gr;
        Collect references to reads that carry the second allele in a set in gr;
    end
    for all genotype records gr in list do
        if PP < PP threshold (0.99) then
            #correct_phase_pir ← 0; #reverse_phase_pir ← 0; max_dst ← 1000;
            look_back(gr, #correct_phase_pir, #reverse_phase_pir, max_dst);
            look_ahead(gr, #correct_phase_pir, #reverse_phase_pir, max_dst);
            if (#correct_phase_pir > 0) OR (#reverse_phase_pir > 0) then
                if #correct_phase_pir > #reverse_phase_pir then
                    validate phase // report number of supporting reads in new PP value;
                else
                    inverse phase // e.g., 0|1 => 1|0 and report number of supporting reads in new
                      PP value;
                end
            end
        end
    end
end
// pir stands for a phase informative read that links 2 heterozygous genotypes
```

For each sample a doubly linked-list of genotype records is created, for ease of access to the previous and next genotypes in the `look_back/ahead` functions. For all genotype records, the associated variant locus is retrieved from the VCF line, and reads are piled-up from the CRAM file at that locus. The reads are filtered through a stringent read filter as defined in Algorithm S2-5. Then for all genotype records, if the PP score is below a threshold (the default threshold is 0.99) then the algorithm tries to rephase the genotype. For doing so, two counters are used (*#correct_phase_pir* and *#reverse_phase_pir*). *pir* stands for **p**hase **i**nformative **r**eads, which are read-pair fragments that overlap two heterozygous genotype loci as depicted in Fig 1. The counters represent the number of reads that validate or invalidate the current phase between the two genotype calls. The `look_back/ahead` functions are called, which check the variant loci backwards and forwards to see if they share sequencing reads with the current variant. They are described in Algorithm S2-2 and S2-3. These algorithms pass through the linked list up to a certain distance threshold (by default 1,000 base pairs, it can be increased, e.g., if long read sequencing data becomes available). They check every variant for shared reads. This is done through Algorithm S2-4 which checks if both alleles of the same estimated haplotype are on the same reads or read-pairs. If so the *#correct_phase_pir* counter is incremented by the number of reads that agree. If the reads show that the alleles are not on the same estimated haplotype the *#reverse_phase_pir* counter is incremented by the number of reads.

Algorithm S2-2: look_back Function

```
Input: Genotype record gr, max_distance
Inout: #correct_phase_pir, #reverse_phase_pir
Function look_back(gr, #correct_phase_pir, #reverse_phase_pir, max_distance):
    prev ← get previous to gr (all the genotype records of a sample are nodes of the same double
      linked-list);
    while prev exists do
        compute distance between prev and gr;
        if distance > max_distance then
            return;
        else
            check_phase(gr, prev, #correct_phase_pir, #reverse_phase_pir);
        end
        prev ← get previous to prev;
    end
```

Algorithm S2-3: look_ahead Function

```
// Same as look_back() but with next instead of previous;
```

Algorithm S2-4: check_phase Function

```
Input: Genotype record gr (to be rephased), grRef (reference)
Inout: #correct_phase_pir, #reverse_phase_pir
Function check_phase(gr, grRef, #correct_phase_pir, #reverse_phase_pir):
    if grRef.PP ≥ PP threshold needed for rephasing (0.99) then
        #correct_phase_pir += Number of reads that map both the first allele of gr and grRef
          (intersection of read sets);
        #reverse_phase_pir += Number of reads that map both the first allele of gr and the second
          allele of grRef (intersection of read sets);
        #correct_phase_pir += Number of reads that map both the second allele of gr and grRef
          (intersection of read sets);
        #reverse_phase_pir += Number of reads that map both the second allele of gr and the first
          allele of grRef (intersection of read sets);
    end
```

Algorithm S2-5: read_filter Function

```
Input   : SAM format Read read (from CRAM file)
Function read_filter(read):
    if read is unmapped OR secondary mapping OR QC failure OR optical/PCR duplicate then
        reject read and return;
    else if read is paired AND (NOT mapped in a proper pair OR mate is unmapped OR both on
      same strand) then
        reject read and return;
    else if read mapping quality < MAPQ50 then
        reject read and return;
    else
        accept read and return;
    end
```

Finally, based on the number of reads that agree or disagree with the current phasing, the genotype phase is updated if needed, and in both cases the number of reads that support the current or new phase is reported in an updated PP-score which is 1.0 + PP + Number of reads, which allow to discern rephased genotypes because their PP is above 1.0, and quantify both the number of reads backing the phasing and the original PP value given by SHAPEIT5, as described in the methods section. The algorithmic complexity of the phase calling algorithm is $\mathcal{O}(MN)$ where M is the number of variant loci and N is the number of samples. In practice, for each sample, only a fraction of all the variant loci in the original VCF/BCF input file are extracted and queried.
