# Supplementary material for: Improving population scale statistical phasing with whole-genome sequencing data
Source: PLoS Genet. 2024 Jul 3;20(7):e1011092. doi: 10.1371/journal.pgen.1011092 (PMC11251608; doi:10.1371/journal.pgen.1011092)
Supplement: S1 Fig — Distributions for the three leftmost classes of variants in Fig 5. In linear scale (top) and logarithmic scale (bottom). (PDF) [file pgen.1011092.s004.pdf]

S4 Figure. SHAPEIT5 phase confidence PP-score distributions on chromosome 21 for the 200,031 samples release of the UK Biobank

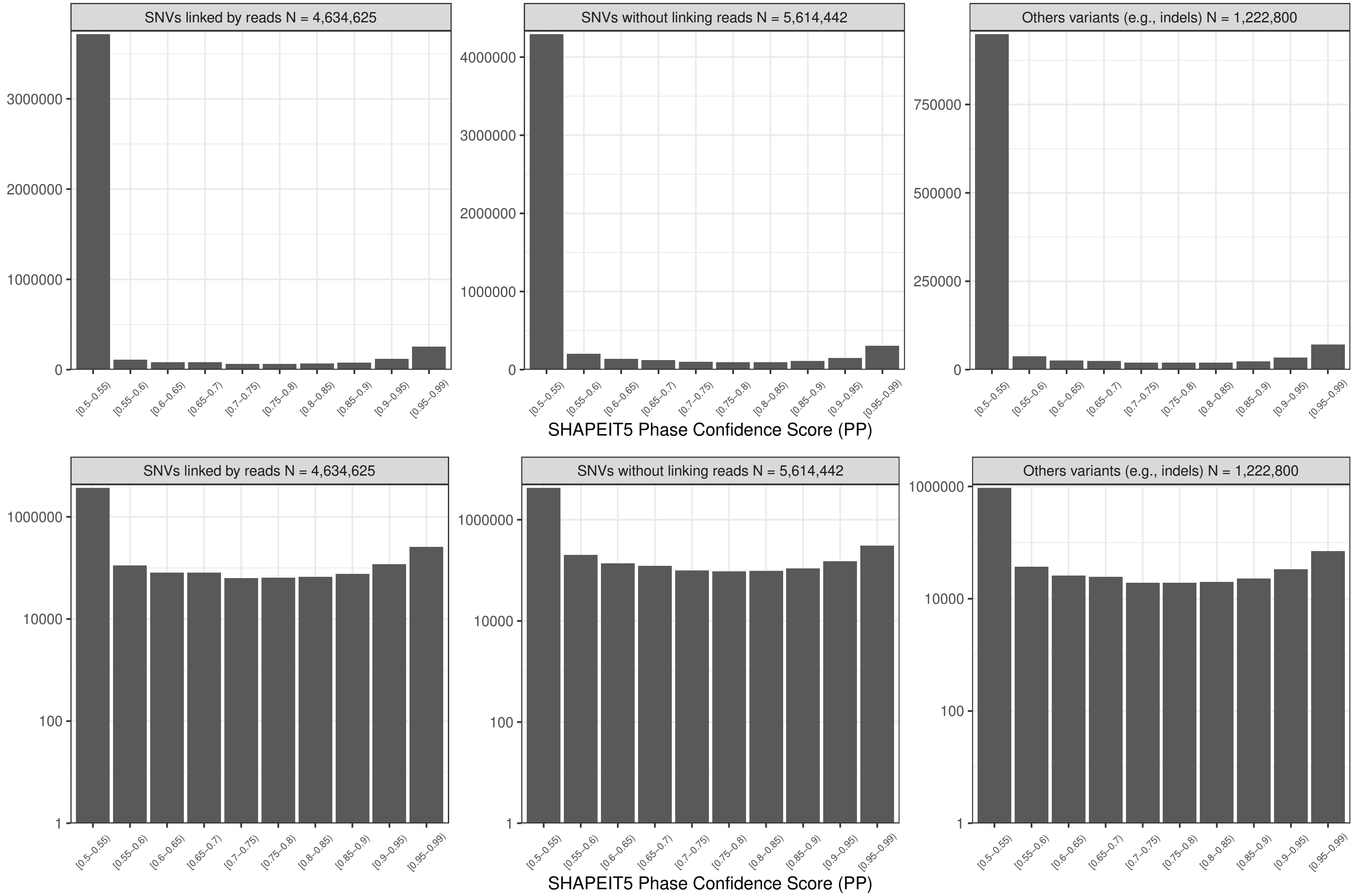

Distributions for the three leftmost classes of variants in Fig 5. In linear scale (top) and logarithmic scale (bottom).
