# Supplementary material for: Improving population scale statistical phasing with whole-genome sequencing data
Source: PLoS Genet. 2024 Jul 3;20(7):e1011092. doi: 10.1371/journal.pgen.1011092 (PMC11251608; doi:10.1371/journal.pgen.1011092)
Supplement: S2 Fig — Top panel: Density of low-PP per 100kB region over chromosome 6. Middle panel: percentage of low-PP variants that share a read-pair with a common variant (green) and percentage of variants switched by the SAPPHIRE method (purple). Bottom panel: Plot of PP value for 100,000 randomly sub-sampled genotypes out of 45,376,832 (black) and average PP score per 100kB region for all genotypes (red). Plotted with karyoploteR [31]. (PDF) [file pgen.1011092.s005.pdf]

S5 Figure. SAPPHIRE on chromosome 6 of the 200k UK Biobank data set

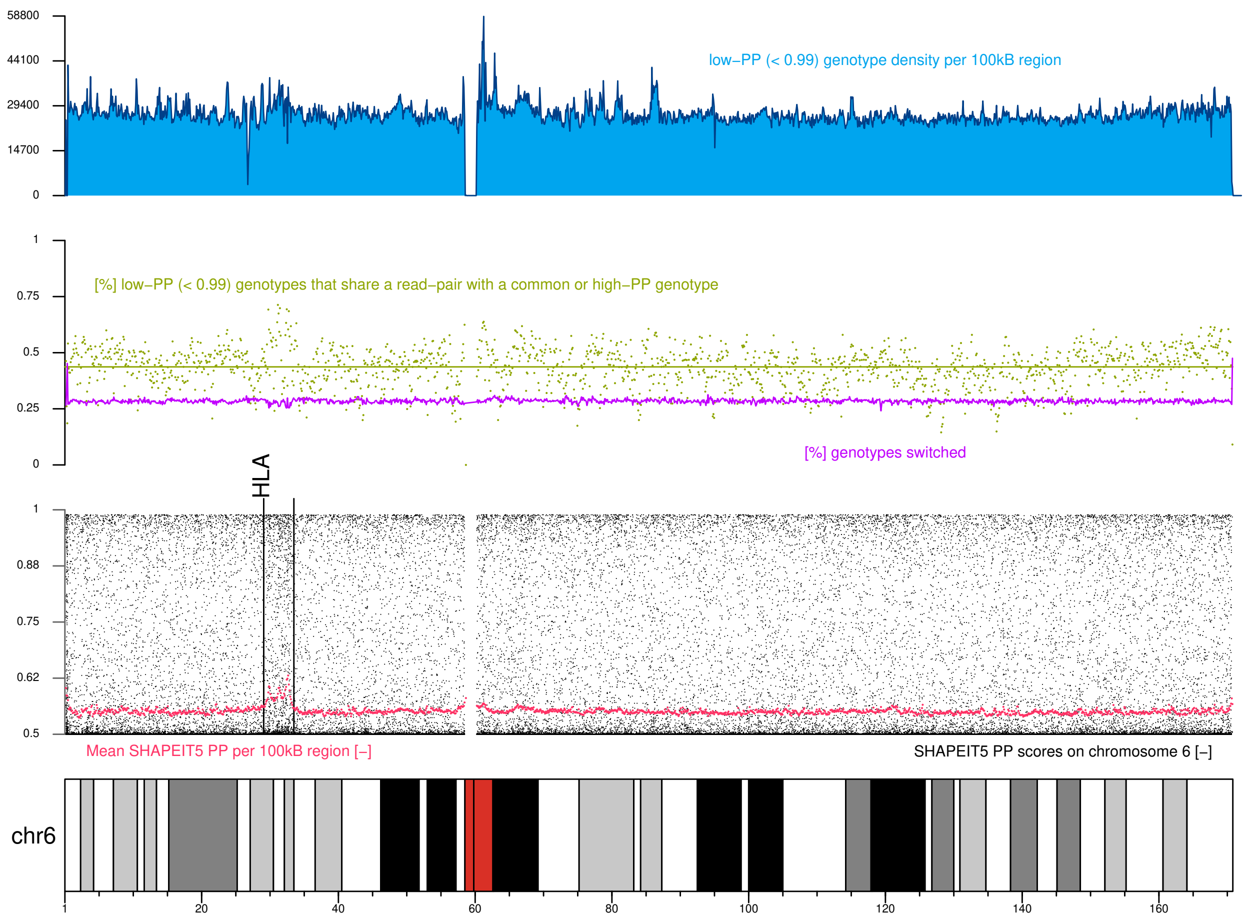

Top panel: Density of low-PP per 100kB region over chromosome 6. Middle panel: percentage of low-PP variants that share a read-pair with a common variant (green) and percentage of variants switched by the SAPPHIRE method (purple). Bottom panel: Plot of PP value for 100,000 randomly sub-sampled genotypes out of 45,376,832 (black) and average PP score per 100kB region for all genotypes (red). Plotted with karyoploteR [1].

[1] Bernat Gel & Eduard Serra. (2017). karyoploteR: an R/Bioconductor package to plot customizable genomes displaying arbitrary data. Bioinformatics, 31–33. doi:10.1093/bioinformatics/btx346
`https://github.com/bernatgel/karyoploteR`
