# Supplementary material for: Improving population scale statistical phasing with whole-genome sequencing data
Source: PLoS Genet. 2024 Jul 3;20(7):e1011092. doi: 10.1371/journal.pgen.1011092 (PMC11251608; doi:10.1371/journal.pgen.1011092)
Supplement: Attachment — Submitted filename: PLOSGenetics_review.pdf [file pgen.1011092.s015.pdf]

Review report: Improving population scale statistical phasing with whole-genome sequencing data

The reviewer acknowledges the effort and time that the authors dedicated to conducting the study and preparing the manuscript for submission. This manuscript presents SAPPHIRE, a novel method for refining haplotype estimates from a statistical phasing method, SHAPEIT5, by leveraging whole-genome sequencing (WGS) data. It targets low-confidence phase calls for re-alignment, enhancing the accuracy of haplotype determination, especially in rare variants. This approach demonstrates significant improvements in phasing accuracy when applied to a large dataset from the UK Biobank.

The topic of this work is interesting and the work is complete. However, I still have some major/minor comments as below.

Major:

- Impact of Sequencing Depth: A discussion on how sequencing depth influences the performance of SAPPHIRE would be informative. Including analyses or guidelines on the minimum sequencing depth required for effective phasing would be valuable for users with varying data qualities.
- Phenotypic Impact Analysis: While the manuscript focuses on the technical improvement in phasing accuracy, a brief exploration or discussion on the potential impact of improved phasing on downstream analyses, such as GWAS or variant interpretation in clinical settings, would enrich the study's relevance.
- Cost of the SAPPHIRE Method: The manuscript reported the saved money of SAPPHIRE when dealing with the UKB WGS dataset. I am also interested in the saved computing time of SAPPHIRE compared to the baseline methods. This would help improve the strength of the manuscript.

Minor:

- The use of blue and pink colors in Figure 1 lacks a clear explanation. Please provide detailed legends or descriptions within the figure caption to clarify what each color represents.
- The manuscript primarily focuses on the performance of SAPPHIRE using whole-genome sequencing data. Given the widespread use of Whole Exome Sequencing (WES) in genetic studies, an evaluation of SAPPHIRE's effectiveness and accuracy on WES data would be valuable.
- How does SAPPHIRE handle different types of genetic variants (e.g., SNPs, indels, structural variants) during the phasing process?
