# Supplementary material for: Improving population scale statistical phasing with whole-genome sequencing data
Source: PLoS Genet. 2024 Jul 3;20(7):e1011092. doi: 10.1371/journal.pgen.1011092 (PMC11251608; doi:10.1371/journal.pgen.1011092)
Supplement: Attachment — Submitted filename: PLOS_Responses_to_reviewers_240517.pdf [file pgen.1011092.s016.pdf]

PLOS Genetics
Responses to reviewers

Dear Editor(s) and Reviewer(s),

We are pleased to submit the revised version of our manuscript “Improving population scale statistical phasing with whole-genome sequencing data”. We appreciate the time and effort taken to read, review, and comment on our manuscript and are grateful for the valuable feedback. The reviewer’s insightful comments have significantly contributed to the enhancement of our manuscript.

Below, we address each of the reviewers' comments, with the original comments in black and our responses in blue. We have made substantial revisions to the manuscript, highlighted in red, including the incorporation of new benchmarks for the SAPPHIRE method on Beagle phased data, as well as new benchmarks on SHAPEIT phased data with minor allele frequency as a proxy for phasing confidence. Additionally, detailed descriptions of the algorithms underlying SAPPHIRE have been added, along with new subsections to clarify points raised by the reviewers.

We sincerely apologize for the delay in submitting the revised version. Due to administrative reasons related to an institutional change, we encountered difficulties accessing the UK Biobank Research Access Platform from March 1st to April 22nd. We appreciate your understanding and patience during this time.

Thank you once again for your time and expertise. We believe that the revisions have significantly improved the quality and clarity of our manuscript, and we hope that you will find the updated version satisfactory for publication.

Sincerely,
Rick Wertenbroek

Reviewer #1: In this manuscript, Wertenbroek et al. proposed an interesting re-phasing method, SAPPHIRE, to correct phasing errors specifically for rare variants, based on the phased output from SHAPEIT5. It takes sequencing read data from CRAM files in addition to the genotype data in VCF/BCF format, and borrows information of a nearby common variant to validate the phasing accuracy. The intrinsic idea of the method is straightforward with a clearly improved phasing accuracy, but the authors should provide more evidence for its broad utility.

The authors would like to thank the reviewer for the insightful comments. SAPPHIRE was designed to improve major phased data sets that will be reused in a multitude of downstream analyses. SAPPHIRE is designed specifically for this, as a post-processing improvement over SHAPEIT5. Phased data releases, for example, in the UK Biobank are used by numerous other researchers and providing the best quality here is paramount as these datasets will be used for years to come (similarly to the 1000 Genome project, which at the time was phased with SHAPEIT2 and is still in use today). The authors will phase the final release of the 500k samples of the UK Biobank with SHAPEIT5 and polish the haplotypes with SAPPHIRE to provide one of the official phased genotype data sets for the UK Biobank. SAPPHIRE allows to generate the best quality possible for these data sets that will be used by researchers for years to come.

Major:
1. I am wondering how the nearby common variants were selected. How the "common" was defined? Was the choice based on allele frequency? Please confirm, SHAPEIT5 does not provide phase accuracy score for common variants, right? SAPPHIRE assumes those common variants were accurately phased, though largely fine, some phasing error may still exist. What if it picks a common variant with phasing error?

Common variants are indeed defined by allele frequency. As with SHAPEIT5, common variants are arbitrarily defined as variants with a minor allele frequency above 0.1%. This has been clarified in the main text. SHAPEIT5 does not give any phase accuracy score to common variants (No PP field provided).

For each heterozygous low-confidence variant, the two nearest previous and next common variants were selected (so 4 common variants, this parameter can be increased during extraction, note that for SAPPHIRE to be able to use them for rephasing they should share a sequencing read or read-pair with the rare variant, which is not always the case). If a common variant has a phasing error, which can happen, the rare variant will be phased on the wrong haplotype, however the phase relation between the rare variant and the common variant will be correct. Depending on the downstream analysis this may be an advantage or a disadvantage. If the relation between the two variants is important (e.g., for compound heterozygous event analysis) then the rephasing is beneficial. Note that the switch error rate will remain the same because by switching the rare variant, the switch error between the rare and common variant disappears and a new switch error is created between the rare variant and the previous variant. So we believe that if a common variant is phased wrong it is still beneficial to have the rare variant to have phase coherence with the common variant, even if both are on the wrong haplotype. If a rare variant is close to more than one common heterozygous variant and they disagree on the phase because one of them is phased wrongly, the rare variant will be rephased according to the biggest number of sequencing reads supporting a given phase. These cases can be reported by SAPPHIRE and could be used to re-evaluate a region if needed.

We have added the definition of common variants in the main text. Added a section on erroneous common variants in methods and provided the exact phase calling (polishing) algorithm in section S3.

2. SAPPHIRE was built based on the phasing accuracy score from SHAPEIT5 by extracting sites with accuracy score < 0.99 for all the rare variants. Though the authors mentioned MAF could be a proxy, they didn't perform analyses based on MAF. I am wondering if the authors could demonstrate the advantages of SAPPHIRE using output from Beagle5 without accuracy score information, especially given that Figure 2 shows Beagle5 achieved the largest phasing error. Will SAPPHIRE similarly achieve improved accuracy?

SAPPHIRE can use MAF as a proxy. We hadn't done the analyses at the time of submission. We have added the benchmarks for BEAGLE and SHAPEIT with MAF as a proxy, we updated the main figure and text. We also provide several extra benchmarks with different values of MAF as a proxy, both for SHAPEIT and BEAGLE. The use of the PP phasing confidence score of SHAPEIT gives the best results, nonetheless results with MAF as a proxy are very close as depicted in new Fig. 10.

3. According to Figure 5, SAPPHIRE could only work for 45.2% of the SNVs which share a sequencing read with

another heterozygous variant. Did the authors assess if there are any differences between the two sets, in terms of both phasing accuracy score from SHAPEIT5 and true phasing accuracy from families? If significant difference exists, the section “assessment of SHAPEIT5 phasing confidence” will be biased, since only a non-randomly selected subset could be assessed.

Both dataset exhibit a similar PP score distribution hinting towards no bias. The main difference between the two sets is the distance to the closest common heterozygous variant. Short-read sequencing data can only link close variants, so we could not evaluate the phase confidence score of all variants through SAPPHIRE. Nevertheless, 45.2% of all SNVs (4,634,625 variants) given a PP<0.99 score by SHAPEIT5, is almost half of all SNVs, therefore we believe that this is still representative and significant. It is hard to theorize as to why this should introduce any major bias. The similarity in the PP distribution between the two sets leads to believe no bias is introduced, randomly subsetting the 4,634,625 variants that share a sequencing read to 10% (463k variants) or 1% (46k variants) still exhibit similar ratios +-3% further pushing the belief that there is no bias. Family data was not used to assess SHAPEIT5 PP score on the 200k data set because this data set includes the trios and duos, therefore introducing bias as SHAPEIT5 makes use of trios and duos during phasing, therefore most variants shared in the duos and trios will have a high PP. We added a supplementary figure (Figure S4) to depict the distributions of PP between the following three classes : “SNVs linked by a read”, “SNVs”, “Other variants (e.g., Indels)”. As the distributions are extremely similar, it does not seem there is a biased selection when taking only into account the variants pairs covered by read fragments.

4. Are there any variations across the genome regarding the phasing accuracy? Does SAPPHIRE also improve phasing accuracy for some highly complicated genome regions, e.g., the HLA region?

Thank you for suggesting this analysis, we have investigated the PP-score distribution over the whole chromosome 6 and SAPPHIRE’s ability to rephase the different regions on chromosome 6, including the HLA region. We have added the section “SAPPHIRE performance in complex genomic regions” in the methods to answer these concerns. SAPPHIRE performs well even in complex regions, even more so in regions with a high number of mutations as this increases the chance of a common/confidently phased genotype to share a read-pair fragment with a low-PP genotype.

Minor:
1. Figure 2: since SAPPHIRE is an phasing enhancement method rather than a phasing method, I suggest the figure legends should change from SAPPHIRE to SHAPEIT5+SAPPHIRE.

Agreed, we updated the figure and we have added Beagle+SAPPHIRE as well.

2. In the section of DNMs, when assessing the relationship between number of DNMs in offspring and paternal/maternal age, the authors only reported results after SAPPHIRE correction. How about before SAPPHIRE correction using only SHAPEIT5 output?

Thank you for pointing this out, while we provided the results for SHAPEIT5 for the parental origin of DNMs (original draft, lines 127-130), we did not provide the relation between DNMs and paternal/maternal age for SHAPEIT5, we have modified figure 4 to now show the distributions for SHAPEIT5 alone and SHAPEIT5 with SAPPHIRE as suggested. Interestingly, this allowed to show that the SHAPEIT5 data alone displayed a spurious negative correlation with the maternal age at conception, which gets corrected through phase polishing. The main text and figure 4 were updated.

3. The authors assessed the performance of SHAPEIT5 confidence score by different bins for variants with score < 0.99. Are the sites with score > 0.99 truly trustworthy? Especially given the last bin with the highest scores also show switch rate 12.5%. Again, this may be biased depending on whether there is significant differences between the two sets of variants (major point 3). Also, the last bin should be [0.95, 0.99) if the authors didn’t evaluate variants with scores > 0.99.

The rare variants above 0.99 were not evaluated, because they were not extracted. Given the results it might be interesting to extract them as we could assume they may indeed not be as trustworthy as expected. As we added extra benchmarks of SAPPHIRE on the SHAPEIT5 data based on MAF as a proxy instead of PP, we can see that the phasing results that rely on common (MAF > 0.001) and high confidence rare variants (PP > 0.99) are better than the results with rephasing based on common (MAF > 0.001) only. We can therefore conclude that those

variants are well phased. We might still assume that the phase confidence of SHAPEIT5 for PP 0.99 could sometimes be a little miscalibrated. This is however, not the focus of this paper. We show that we can use SAPPHIRE to evaluate phasing confidence scores, but this is not a thorough evaluation of SHAPEIT5 phasing confidence scores nor the main goal of SAPPHIRE.

We have analyzed the three classes of variants, they exhibit an extremely similar PP distribution, we reported this in supporting information S4 Figure, this gives us confidence that there is no bias between the three classes, also randomly subsampling the data set has shown similar results.

Thank you for spotting the last bin designation, it should indeed be [0.95, 0.99) and this has been updated in the figure.

Reviewer #2: Wertenbroek et al proposed an innovative statistical phasing method based on whole-genome sequencing data. I think the method looks valid considering that they have compared their software with other software in literature, and applied to PB-level big data. However, the methodology description is unclear such that I have a lot of questions after reading the manuscript.

The authors would like to thank the reviewers for the insightful comments. We hope to answer the reviewers questions and have added extra sections to clarify.

Major Comments:

1. SAPPHIRE is the author's proposed software. However, I did not find the description of statistical methods used in the software. What is the innovation and advantage of SAPPHIRE? I only find they make corrections on phasing error, but do not know whether this correction improves statistical phasing, or just via post simple adjustment. Please specify in detail the methods used in the software.

SAPPHIRE does not rely on statistical methods, except counting the number of sequencing reads that validate or correct (switch) the phase. We improve phased data sets that were produced by statistical phasing methods, we do not improve statistical phasing methods themselves. SAPPHIRE is indeed a “simple” post processing step that improves the quality of statistically phased data sets. SAPPHIRE helps to phase rare variants through the use of sequencing data because statistical methods cannot phase these variants correctly due to the lack of statistical power. We added two extra sections describing the extraction and phase calling algorithms in detail. The algorithms are presented in pseudo-code and details are discussed in Sections S2 Algorithm. Heterozygous variant extraction from VCF/BCF file and S3 Algorithm. Phase calling (polishing). These descriptions combined with the fact that SAPPHIRE is open-source and publicly available should allow researchers to understand the mechanisms underlying the SAPPHIRE method.

2. It is important for the authors to report computational time for different computational nodes. But I did not have full picture of computational time. Can the authors specify what growth pattern of computational complexity with respect to sample size. The current version did not provide any sentence on it.

Thank you for suggesting this, we added the computational complexity of both algorithms in the new sections S2 and S3.

The details of computational costs of the different parts of the SAPPHIRE pipeline are given in supplementary S1 Table. Extraction and Update cost scale linearly with number of samples * variants, the number of variants can be estimated from the different chromosomes sizes. As for the growth pattern of phase calling, this is linear with the number of extracted variants and samples. Because samples are independent, the algorithm can be parallelized to a high degree and therefore cost (time*price per node) becomes the main metric, but time is also reported.

3. I think SAPPHIRE may do post-phasing adjustment, or how they make use of sequencing data? Details are needed.

Indeed we do post-phasing adjustment (described here as phase polishing). The use of sequencing data is described in the methods under “Phase calling and polishing of extracted sites” and “Read quality filtering”. We do a pileup, through HTSLIB, this is the same method as the BCFTools pileup method. References are given and a sketch of the read-based validation is given in Fig. 1B. The read filtering is different than BCFTools and has its own section under “Read quality filtering”, we use only reads that map with very high quality so as to be sure not to degrade the phasing. We chose to not “touch” variants that are on reads that do not map well or are of low quality. In order to clarify we have fully described the phase calling (polishing) algorithm in section S3. References to the pile-up method are given in the main text.

4. The real data part is ok. I know it is a big data, and still there is lack of validity.

The UK Biobank provides one of the biggest data set publicly available. They provide not only the population variant genotype calls but also the whole genome sequencing data for each sample which provides validation. We used the sequencing data of trios (child and both parents) and duos (child and either father or mother) to validate the phase calls of SAPPHIRE. We use the same validation procedures as in the SHAPEIT5 paper [a].

[a] “Hofmeister RJ, Ribeiro DM, Rubinacci S, Delaneau O. Accurate rare variant phasing of whole-genome and whole-exome sequencing data in the UK Biobank. Nature Genetics. 2023; p. 1–7.”.

5. What is the evaluation criteria in comparing multiple software, though Fig 5 and 6 seems to be acceptable.

We compared the phasing accuracy of SHAPEIT5 and BEAGLE against SHAPEIT5+SAPPHIRE and now BEAGLE+SAPPHIRE based on switch error rate, this is based exactly on the comparison method described in [a] which is referenced in the main text. The exact same software and data from that paper was used. Our new figures (Fig 2 and 10) overlap with the figure in [a] and can be directly compared. We also added numerous new benchmarks with MAF as a proxy for PP, figure 2 now also includes SAPPHIRE on Beagle phased haplotypes and new figure 10 includes all benchmarks for different MAF values.

[a] "Hofmeister RJ, Ribeiro DM, Rubinacci S, Delaneau O. Accurate rare variant phasing of whole-genome and whole-exome sequencing data in the UK Biobank. Nature Genetics. 2023; p. 1–7.".

6. What is the new methods for the software.

The new method is an extremely fast and cost effective phase polishing method that improves the phasing accuracy of large population scale datasets phased through statistical phasing. The method is simple: for each pair of low-confidence/high confidence common variants, check if the sequencing data (ground truth) agrees with the phasing, if not rephase the low-confidence variant according to the sequencing data. Prior methods could not scale efficiently and did not provide a pipeline that could improve an already phased dataset of this magnitude. There is no other method in the state-of-the-art capable of generating phased data sets of the quality of SHAPEIT5+SAPPHIRE at a tractable cost and processing time. The resulting phased data of this method is of lower error rate than the state-of-the-art phasing methods and for the moment there are no scalable solutions to achieve this quality. We hope that the new sections S2, S3 allow to clarify the methods used in SAPPHIRE.

7. I did not get the idea of SAPPHIRE. The authors should try to write the methods and derivation. Without clear methodology, I do not know whether their results are stable.

We understand the method might not have been described in the clearest way, we have added sections S2 and S3 describing the algorithms in detail, discussing details and overall complexity. Results are "stable" because there is no use of randomness in the software. (Also the added section on SAPPHIRE on chromosome 6 demonstrates that SAPPHIRE works even in complex regions e.g., HLA region)

8. Is it possible to conduct simulation studies, for example, using COSI software?

Although it is possible to conduct simulation studies, for example by simulating the variants through coalescent simulation with COSI [a] or msprime [b], this would only generate the haplotypes and not the sequencing data. SAPPHIRE relies on sequencing data to correct phasing errors. Generation of sequencing data through simulation is in theory possible but would be extremely costly in terms of processing and storage for 100'000s to millions of samples. Simulation of WGS of this magnitude is simply too costly and may not be very useful as big real data sets exist. In this paper we both use the 150k and 200k releases of the UK Biobank to demonstrate the effectiveness of SAPPHIRE, which improves the phasing accuracy, measured through switch error rate as described in [c]. When available we will apply SAPPHIRE on the 500k release of the UK Biobank.

[a] "Cosi2: an efficient simulator of exact and approximate coalescent with selection", by Ilya Shlyakhter Pardis C. Sabeti and Stephen F. Schaffner, Bioinformatics (2014) 30 (23): 3427-3429. doi: 10.1093/bioinformatics/btu562 .

[b] Baumdicker, Franz, et al. "Efficient ancestry and mutation simulation with msprime 1.0." *Genetics* 220.3 (2022): iyab229.

[c] Hofmeister RJ, Ribeiro DM, Rubinacci S, Delaneau O. Accurate rare variant phasing of whole-genome and whole-exome sequencing data in the UK Biobank. Nature Genetics. 2023; p. 1–7.

9. Is the phasing only for SNPs, or it allows genetic variables beyond SNPs, for example multiple alleles, copy numbers, etc. The authors do need to describe their methods in a clear way. Currently, it seems like a black box, which makes evaluation very difficult.

This is described in the methods section lines 269-270 (in original draft) : "Extracted heterozygous sites are filtered on variant type and only single nucleotide variants (SNVs) are processed, as shown in Fig 5.", SAPPHIRE does only rephase SNVs (SNPs). We understood that the methods section did not provide enough internal

details so we have provided a detailed description of the phase calling (polishing) algorithm in section S3 as pseudo-code so that it does not look like a “black box”, furthermore SAPPHIRE is fully open-source (see section “Software availability” lines 417-421 (in original draft)) and anyone interested in the exact implementation details is free to do so. We now also added sections S2 and S3 that describe the algorithms used in SAPPHIRE. The authors hope that with these new sections the reader will be able to understand the internal mechanisms of SAPPHIRE and would like to thank the reviewer for suggesting these modifications.

Minor Comments

1. I think the manuscript will improves a lot if the authors can write down the methods used in detail.

Thank you for suggesting this, we have described the two main steps of the SAPPHIRE pipeline algorithms with descriptive text and details in the new sections S2 and S3.

Reviewer #3: The review has been uploaded as an attachment.

Review report: Improving population scale statistical phasing with whole-genome sequencing data

The reviewer acknowledges the effort and time that the authors dedicated to conducting the study and preparing the manuscript for submission. This manuscript presents SAPPHIRE, a novel method for refining haplotype estimates from a statistical phasing method, SHAPEIT5, by leveraging whole-genome sequencing (WGS) data. It targets low-confidence phase calls for re-alignment, enhancing the accuracy of haplotype determination, especially in rare variants. This approach demonstrates significant improvements in phasing accuracy when applied to a large dataset from the UK Biobank.

The topic of this work is interesting and the work is complete. However, I still have some major/minor comments as below.

The authors would like to thank the reviewer for the insightful comments. We have extended our analyses and now included Beagle+SAPPHIRE results as well as the original SHAPEIT5+SAPPHIRE results.

Major:

- Impact of Sequencing Depth: A discussion on how sequencing depth influences the performance of SAPPHIRE would be informative. Including analyses or guidelines on the minimum sequencing depth required for effective phasing would be valuable for users with varying data qualities.

The sequencing depth will impact SAPPHIRE in the following ways; First, the sequencing depth will increase the probability of a sequencing read/read-pair to cover both the rare variant and common variant, without at least one sequencing read/read-pair linking both variants, nothing can be done. So at least one read/rad-pair should cover both in order to extract the phase information. The more reads cover the variant pair, the more certain the phase will be. SAPPHIRE reports this number. Second, the read depth will increase the chance of a good quality read/read-pair to cover the variants, quality matters for the filter we use. So in both ways the more coverage the more chances there are to cover both variants. SAPPHIRE will be able to use any coverage, even low-coverage could be used to maybe rephase some variants, the processing time of SAPPHIRE will decrease as the sequencing files will be smaller and regions without coverage are rapidly skipped.

Thank you for suggesting this discussion, we have added references on the sequencing depth in our data set, mean and minimum coverage numbers in the main text. (32.5x / 23.5x), we also added a discussion on low coverage WGS and WES.

- Phenotypic Impact Analysis: While the manuscript focuses on the technical improvement in phasing accuracy, a brief exploration or discussion on the potential impact of improved phasing on downstream analyses, such as GWAS or variant interpretation in clinical settings, would enrich the study's relevance.

We agree, that is why we explored the parental origin of de-novo mutations, GWAS could be another application, however since most rephased variants are very rare (more than 50% have a minor-allele-count with less than 20 (< 0.0001 MAF)) and a majority are singletons, if these variants have significance for GWAS, they will show an effect regardless of their phase as they are heterozygous, with very low statistical power so the effect must be very discernable (i.e., show independently of phase). The phase would matter for compound heterozygous events in GWAS, for example a deleterious mutation on a given gene on both chromosomes at different loci, rather than the two on the same chromosome. Although it would be worthwhile to add a phenotypic impact analysis for compound heterozygous events, this is beyond the scope of this paper.

- Cost of the SAPPHIRE Method: The manuscript reported the saved money of SAPPHIRE when dealing with the UKB WGS dataset. I am also interested in the saved computing time of SAPPHIRE compared to the baseline methods. This would help improve the strength of the manuscript.

Agreed, the computational time saving is also important, and cost is not always the main factor. We have estimated the baseline methods time in section “Cost estimation of read based phasing”. Since both baseline (Whatshap) and SAPPHIRE methods are sample independent, it is possible to parallelise on a per sample basis, this can reduce processing time tremendously depending on the number of compute nodes used. On chromosome 22 baseline for the phasing is about 14 min for a sample, SAPPHIRE is about 10 seconds (7h14 phase calling for 10’000 samples on 4 cpu instance). Therefore, because both methods can be parallelized on a per sample basis, cost was reported as CPU hours and not hours. Finally, for both methods the creation of the final BCF/VCF will take about the same amount of time, because all samples and all variants need to be written to a single file. We have added the big-O time complexity of the methods in the supplementary sections S2 and S3.

Minor:

- The use of blue and pink colors in Figure 1 lacks a clear explanation. Please provide detailed legends or descriptions within the figure caption to clarify what each color represents.

The pink and blue colors are used to represent the two real haplotypes, they have no other meaning here, they can be interpreted as maternal and paternal haplotypes, a note has been added in the figure description to clarify this.

- The manuscript primarily focuses on the performance of SAPPHIRE using whole-genome sequencing data. Given the widespread use of Whole Exome Sequencing (WES) in genetic studies, an evaluation of SAPPHIRE’s effectiveness and accuracy on WES data would be valuable.

We agree that this information could be valuable for projects that have access to whole-exome sequencing data and have added a discussion in the main text. However, WES covers only about 2% of the entire genome and many low-confidence variants will fall outside of this region. Also, the fragment length of WES inserts is usually smaller than WGS resulting in less variants sharing a read. Also about 80% of the exons on each chromosome are < 200bp [r] Therefore, improvement of the dataset with whole-exome will be marginal. We have described this in the section “Number of rare variants that can be rephased through SAPPHIRE” as it is interesting to note.

[r] Sakharkar MK, Chow VT, Kangueane P. Distributions of exons and introns in the human genome. In Silico Biol. 2004;4(4):387-93. PMID: 15217358.

- How does SAPPHIRE handle different types of genetic variants (e.g., SNPs, indels, structural variants) during the phasing process?

SAPPHIRE only handles SNVs (SNPs) as described in the methods section, lines 269-270 (of the original draft). It does not rephase indels and structural variants. The extraction process of the SAPPHIRE pipeline still will extract non SNV variants but the phase caller will not evaluate these variants for the moment. non-SNV variants require careful alignment of the reads and a more complex rephasing algorithm. This is left in the works for a future version of SAPPHIRE. Most variants are SNV (89.4% for example in chr21 with the 200k dataset) therefore SAPPHIRE was designed to rephase SNVs first. This choice was also motivated by the fact that SAPPHIRE is a phase polishing method and should improve phasing, not deteriorate it, because of the complexity of SVs a rushed implementation could decrease phasing quality, hence the very strict filtering of reads and only rephasing of SNVs. As SAPPHIRE is open-source, it could be modified by others to include an indel / SV phase caller.

Have all data underlying the figures and results presented in the manuscript been provided?
Large-scale datasets should be made available via a public repository as described in the *PLOS Genetics* data availability policy, and numerical data that underlies graphs or summary statistics should be provided in spreadsheet form as supporting information.

Reviewer #1: **No:** Lack of supplementary tables for some numerical results; no data availability section

Reviewer #2: Yes

Reviewer #3: None

We had filled the "Data Availability" section in the submission website :

"The UK Biobank data set is a combination of Imaging, Genetics, Health linkages, Biomarkers, Activity monitors, Online questionnaires, and Samples from 500,000 participants, each with their own data sharing policies and restrictions. These restrictions do not allow the data set to be shared in a completely unrestricted way. However, readers may apply for access at https://www.ukbiobank.ac.uk/enable-your-research/apply-for-access"

This statement was also added to the document for peer review as it did not appear.

PLOS authors have the option to publish the peer review history of their article (what does this mean?). If published, this will include your full peer review and any attached files.

If you choose "no", your identity will remain anonymous but your review may still be made public.

Do you want your identity to be public for this peer review? For information about this choice, including consent withdrawal, please see our Privacy Policy.

Reviewer #1: No

Reviewer #2: No

Reviewer #3: No
